# Association between neutrophil-lymphocyte ratio and lymph node metastasis in gastric cancer: A meta-analysis

**DOI:** 10.1097/MD.0000000000029300

**Published:** 2022-06-24

**Authors:** Krishna Kotecha, Animesh Singla, Philip Townend, Neil Merrett

**Affiliations:** a Department of Upper Gastrointestinal Surgery, Royal North Shore Hospital, NSW, Australia; b Department of Vascular Surgery, Royal North Shore Hospital, NSW, Australia; c Department of Upper Gastrointestinal Surgery, Gold Coast University Hospital, Southport, QLD, Australia; d Department of Upper Gastrointestinal Surgery, Bankstown Hospital, Bankstown, NSW, Australia; e School of Medicine, Western Sydney University, Campbelltown, NSW, Australia.

**Keywords:** cancer diagnosis and workup, gastric cancer, surgical oncology

## Abstract

**Introduction and Aim:**

The prognostic role of neutrophil to lymphocyte ratio (NLR) has been explored extensively in the literature. The aim of this meta-analysis was to evaluate the link between NLR and lymph node metastasis in gastric cancer. A method for increasing specificity and sensitivity of pre-treatment staging has implications on treatment algorithms and survival.

**Search Strategy:**

The relevant databases were searched as per the Preferred Reporting Items for Systematic Reviews and Meta-Analyses flowchart. After selection, 12 full text articles that met the inclusion criteria were included for quantitative analysis. 2 × 2 squares were generated using lymph node positive/negative, and NLR high/low data. The effect size for each study was calculated using the DerSimonian–Laird random effects model. *P* values were calculated using the chi-square method. Finally publication bias was evaluated. All statistics were calculated using R Studio.

**Results:**

Meta-analysis showed a 1.90 times (odds ratio, with 95% CI 1.52–2.38) increase in risk of positive lymph node status with high neutrophil to lymphocyte ratio. This has significant implications for cancer screening and staging, as NLR is a highly reproducible, cost-effective, and widely available prognostic factor for gastric cancer patients. Additionally, high or low NLR values may have implications for management pathways. Patients with lymph node metastasis can be offered neoadjuvant chemotherapy, avoiding salvage therapy in the form of adjuvant chemoradiotherapy, which is poorly tolerated.

**Conclusion:**

This meta-analysis shows an association between NLR and positive lymph node status in gastric cancer patients with implications for staging, as well as preoperative personalisation of therapy.

## 1 Introduction

Gastric cancer (GC) is one of the most common neoplasms worldwide and is associated with poor prognosis with treatment pathway dependent on tumor staging.^[1]^ Patients with early gastric cancer with no or limited nodal involvement, may be suitable for upfront surgical resection possibly with further adjuvant therapies. More advanced tumors including those with more significant lymph node disease are treated with surgery combined with neoadjuvant and/or adjuvant chemotherapy or chemoradiotherapy, whereas patients with distant metastases (M1) are typically managed non-surgically. TNM staging and categorization reflects the biological behavior and phenotype of a tumor, which is mediated by the systemic inflammatory response. Neutrophils, derived from the common myeloid progenitor, form part of the innate immune system. Lymphocytes (B and T cells), derived from the common lymphoid progenitor, form part of the adaptive immune system. Lymphocytopenia is an impaired cell mediated immune response, whereas neutrophilia is representative of a systematic inflammatory response. Thus the serum neutrophil-lymphocyte ratio (NLR) reflects a balance between activation of antitumor immune function and pro-tumor inflammatory pathways. The aim of this meta-analysis is to show that pre-treatment NLR is associated with increase in risk of lymph node (LN) metastasis in GC. As a simple, inexpensive preoperative investigation, this would be a valuable addition to the existing diagnostic pathway, as it can improve accuracy of staging and prognosis. Patients who have LN spread may benefit from neoadjuvant therapies according to current guidelines, as adjuvant therapy is not well tolerated in western populations. Patients with a higher risk of metastatic disease should receive individualized treatment, as patients with the same TNM stage can have different clinical outcomes.^[2,3]^ Finally, patients with high NLR should be recognized as a high risk group in terms of recurrence, thereby altering the pattern of post-treatment follow-up.

## 2 Methods

### 2.1 Literature search

This systematic review with meta-analysis was performed in accordance with the Preferred Reporting Items for Systematic Reviews and Meta-Analyses statement.^[4]^ The PubMed, Embase, and Cochrane library were searched for all articles without restriction using the following search criteria: (“neutrophil-lymphocyte ratio” OR “neutrophil-to-lymphocyte ratio” OR “NLR” OR “neutrophils” OR “lymphocytes”) AND (“gastric cancer” OR “gastric adenocarcinoma”) and (“lymph node” OR “lymphadenopathy” OR “metastasis” OR “metastatic spread”). This search strategy was performed until no relevant article was found. Grey literature searches were also performed. Overlapping or duplicate data was excluded. The 3 databases were searched from inception to January 12, 2021. All searches were performed independently by 2 authors. As a meta-analysis of published data, no ethics board approval was required.

### 2.2 Article selection

**Figure 1:**
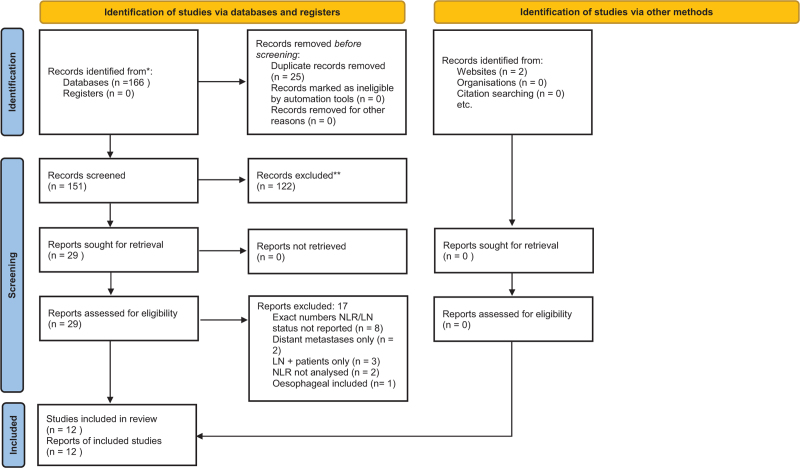
PRISMA flowchart. From: Page MJ, McKenzie JE, Bossuyt PM, Boutron I, Hoffmann TC, Mulrow CD, et al. The PRISMA 2020 statement: an updated guideline for reporting systematic reviews. BMJ 2021;372:n71. doi: 10.1136/bmj.n71. For more information, visit: http://www.prisma-statement.org/. PRISMA = Preferred Reporting Items for Systematic Reviews and Meta-Analyses.

The article selection process is summarized in the Preferred Reporting Items for Systematic Reviews and Meta-Analyses flowchart (Fig. 1). The population of interest was patients with a diagnosis of gastric cancer who had pre-treatment neutrophil and lymphocyte count recorded, and the comparison of interest was patients with lymph node metastases.

Studies were included if they provided the following data about gastric cancer patients:

(1) Pre-treatment NLR.
(2) Number of patients in low-NLR and high-NLR groups, with the NLR cutoff defining these groups.
(3) Number of patients in lymph node positive and negative groups.

Studies were excluded if;

(1) Insufficient data was provided, for example, NLR cut off or number of cases with lymph node involvement not provided.
(2) Blood counts were not derived from the patients’ pre-treatment investigations.
(3) They were abstracts, letters, editorials, expert opinion reviews, case reports.
(4) They were not in English.

### 2.3 Data extraction

Data were extracted independently and manually by 2 investigators. For each study, the following data were extracted; year of publication, author name, country of origin, study design and setting, total number of cases, demographic features, clinicopathological characteristics (sex, age, tumor location, Lauren classification, TNM stage), NLR cut off, number of cases with elevated and reduced NLR, number of cases with positive and negative lymph node status, ages of subjects. No other variables were sought. Missing data from the above fields were marked as such.

### 2.4 Quality assessment and risk of bias

All eligible articles were evaluated independently by 2 reviewers for risk of bias according to the Newcastle Ottawa Scale (NOS) (see Supplemental Digital Content Appendix 1; Table containing NOS scoring system of study quality).

### 2.5 Statistical analysis

**Figure 2:**
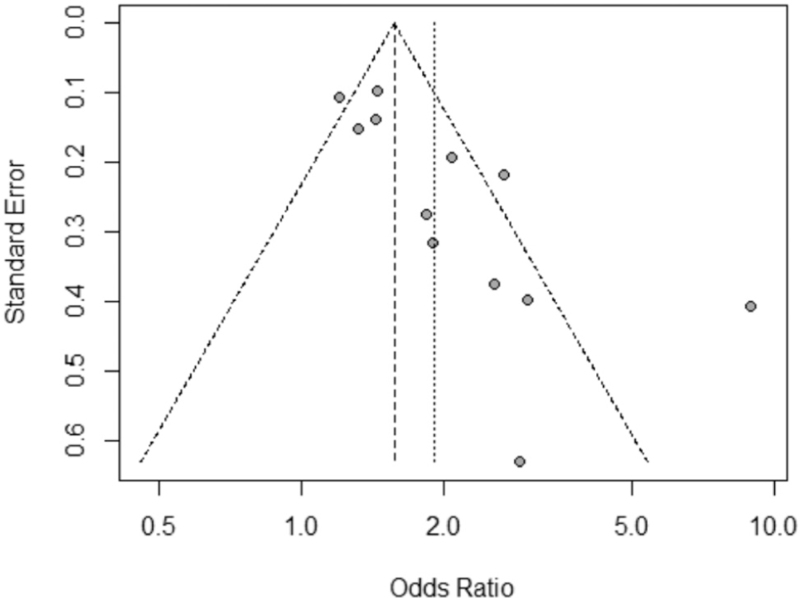
Funnel plot in assessment of publication bias.

For this meta-analysis, *I*^2^ was used to evaluate heterogeneity, with value >50% representing possibility of substantial heterogeneity. If the *I*^2^ value exceeded 50%, the effect size (odds ratios and 95% confidence interval) for each study was calculated using the DerSimonian–Laird random effects model. 2 × 2 squares were generated using lymph node positive/negative, and NLR high/low data. This data is available in Appendix 2 (Supplemental Digital Content Appendix 2: 2 × 2 squares containing extracted data from studies). *P* values were calculated using the chi-square method. Finally publication bias was evaluated by visual inspection of the funnel plot for symmetry, and the Harbord^[5]^ test (see Fig. 2), with *P* < .05 indicative of possible publication bias. All statistics were calculated using R Studio.^[6]^

## 3 Results

### 3.1 Search results

A total of 168 references were generated: PubMed (n = 96), Embase (n = 54), Cochrane Library (n = 16), grey literature, and references (N = 2). Following the eligibility criteria, only 97 were eligible studies. After screening, 29 studies were reviewed and analyzed. Eight articles^[7–14]^ were excluded as they did not provide the exact number of patients who were NLR positive or negative, and lymph node positive or negative. One article combined platelet-lymphocyte ratio and neutrophil-lymphocyte ratio^[15]^; 2 articles included patients with distant metastases only^[16,17]^; 3 articles included lymph node positive patients only^[18–20]^; 2 articles did not analyze neutrophil-lymphocyte ratio as predictive factors for gastric cancer,^[21,22]^ and 1 article included esophago-gastric cancers.^[23]^ Finally, we identified 12 full text articles^[10,24–34]^ that met the inclusion criteria for quantitative analysis.

### 3.2 Characteristics of studies

The meta-analysis included 9401 patients, summarized in Table 1. Five studies were identified as high quality (NOS >6).

**Table 1:** Summary of results.

Year	First author	Country	Study design	Patients (n)	Age median	NOS	Cut off value NLR
2018	Zhang LX	China	R	904	N/A	**7**	2
2016	Pang W	China	R	927	63	**7**	1.59
2017	Song S	China	P	1990	62	**5**	2.10
2015	Yu L	China	R	291	N/A	**8**	3.5
2015	Kim EY	S. Korea	P	1986	58.2	**6**	3
2014	Jiang N	China	R	377	64	**6**	1.44
2015	Hsu JT	Taiwan	R	1030	N/A	**6**	3.44
2010	Ubukata H	Japan	R	157	65	**6**	5
2010	Shimada H	Japan	R	1028	65	**5**	4
2018	Zhang Y	China	P	182	65	**7**	2.88
2018	Mori M	Japan	R	100	66	**6**	1
2019	Kosuga T	Japan	R	429	67	**7**	1.6
		**9401**					

NLR = neutrophil to lymphocyte ratio, NOS = Newcastle Ottawa Scale, P = prospective, R = retrospective.

### 3.3 Meta-analysis

The results are displayed with forest plot in Table 2. This showed a 1.90 times pooled OR (95% CI 1.52–2.38) of lymph node metastasis in patients with high neutrophil to lymphocyte ratio. High neutrophil to lymphocyte ratio was defined as a value above the cut-off set for each individual study.

**Table 2:**
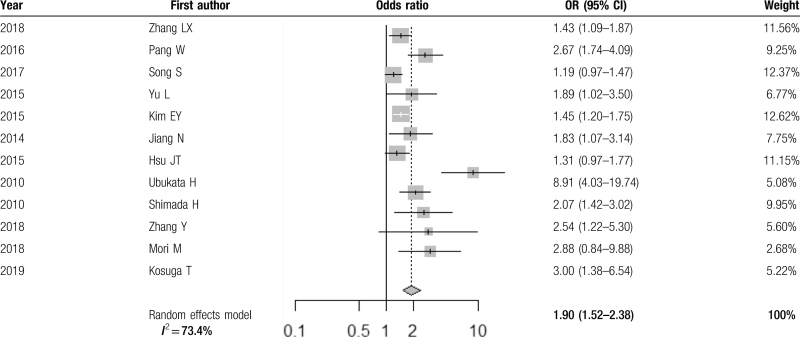
Random effects model showing pooled odds ratio.

### 3.4 Heterogeneity

Significant heterogeneity among studies was found *I*^2^ = 73.4%, *Q* = 41.33 (*P* < .01), and therefore the random effects model was used. After performing sensitivity analysis, we found that the study by Ubukata et al^[31]^ contributed more to the heterogeneity—on exclusion analysis, heterogeneity fell to 55.6%, and yet the overall pooled OR was 1.68 (95% CI 1.41–2.00). (See Table in Supplemental Digital Content, Appendix 3: exclusion analysis of studies). To investigate the heterogeneity, metaregression was also performed, including covariates (publication country, study size, publication year, NLR cut-off value).

### 3.5 Biases

**Figure 3:**
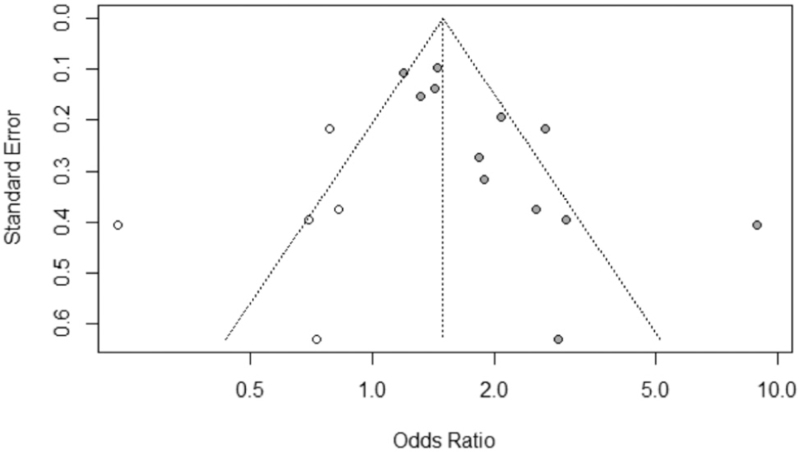
Funnel plot following trim-and-fill estimator correction for asymmetry.

A funnel plot (Fig. 2) demonstrated asymmetrical dispersion of LN metastases. The Harbord test revealed a possibility of publication bias (*P* < .05). This result must be interpreted with caution, given the low number of studies (10) included in this meta-analysis. Additionally, type II error may exist due to the limited number of publications. The trim-and-fill estimator method^[35]^ to correct for funnel plot asymmetry. These results are available in Table 3 (with filled results in Fig. 3), and show a pooled OR of 1.45 (95% CI 1.11–1.80), a positive association between NLR and LN positive status in patients with GC.

**Table 3:**
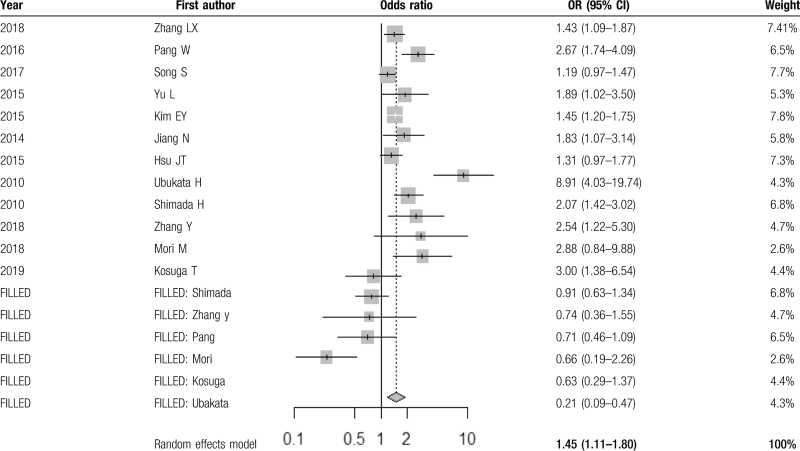
Trim-and-fill method to adjust for funnel plot asymmetry.

Given the majority of studies originated in Asia, there is the possibility of location bias. There were very few missing results reported in the studies.

## 4 Discussion

### 4.1 The role of inflammation in cancer growth and spread is well established

Markers such as C-reactive protein, neutrophil-lymphocyte ratio (NLR) and platelet-lymphocyte ratio (PLR), represent a non-specific inflammatory response to tumor hypoxia, tissue injury, and necrosis.^[36]^ The ability of gastric cancers to act aggressively and metastasize is dependent on the intrinsic characteristics of the tumor cells themselves, as well as the cancer microenvironment.^[37]^

Several models of cancer formation have been described. Reactive oxidative species and cytokines formed as part of an inflammatory disease state can initiate oncogenesis, and conversely,^[38]^ existing cancer can provoke an inflammatory response that allows the genetic transformation of a low grade malignancy into a high grade one.^[39]^ These genetic influences are upregulated through transcription factors such as nuclear factor kB, signal transducer, and activator of transcription 3 and hypoxia inducible factor 1a.^[40]^ As part of this process, downregulation of the adaptive immune system creates a self-perpetuating tumor stimulating environment.

Several cell mediated processes are involved in tumor pathogenesis;

(1) Neutrophils play a key role, as they are involved with the initial and subsequent inflammatory response. Neutrophil associated inflammatory mediators include vascular endothelial growth factor and matrix metalloproteinases, which inhibit the antitumor effects of helper T-cells (CD4), cytotoxic T-cells (CD8), and natural killer (NK) cells.^[36]^ They also stimulate tumor angiogenesis via remodeling of the extracellular matrix.^[41]^
(2) In addition to neutrophils, platelets have several functions in tumor pathogenesis. Although the prognostic role of platelet count, and platelet-to-lymphocyte ratio have been extensively discussed in the literature, they are not the focus of this study. In the tumor microenvironment, platelets facilitate tumor adhesion to vascular endothelium through the formation of tumor thrombi, thus protecting them against immune clearance.^[42]^ Several secretory chemokines (interleukins 1 and 6, tumor necrosis factor α, thrombospondin, leukemia inhibitory factor, and endostatin) are directly released by platelets, potentiating metastatic spread.^[9]^ In particular, the aforementioned vascular endothelial growth factor, along with platelet derived growth factor aid in tumor angiogenesis and metastasis.^[43]^ Additionally, a high platelet count causes a lymphocytopenia, and thus a hypoimmune response linked to lymphocyte-mediated anti-tumor activity at the cellular level, interlinking high platelet count to raised NLR. A robust lymphocyte response is a major factor in the suppression of cancer progression, and therefore, a relative lymphocytopenia leads to a blunted lymphocytic (T_4_H and T_8_) anti-tumor response, raising NLR further.
(3) NLR can therefore be considered as the balance between pro tumor inflammatory status and immune mediated tumor suppression. Patients with elevated NLR have a relative lymphocytopenia and neutrophilic leucocytosis, shifting the balance in favor of protumor inflammation. This systemic inflammation is associated with functional and nutritional decline,^[44]^ a poor oncological outcome,^[45]^ and poorer survival in gastric cancer.^[46]^

### 4.2 Elevated NLR is a proven negative prognostic factor in many cancers, including gastric cancer

NLR has been shown to be a negative prognostic factor in other cancers, including breast, colorectal, esophageal, liver, melanoma, ovarian, pancreatic, and prostate.^[47]^ As a result, there have been numerous novel studies incorporating NLR into preoperative staging. Huang et al^[48]^ recommended a preoperative COCT-NLR (a combination of NLR and contrast enhanced computed tomography) to detect LN metastasis in non-small cell lung cancer patients, with a high sensitivity (70.59%) and specificity (74.89%). Similarly, Ertas et al^[49]^ showed an independent association between both preoperative NLR and PLR and lymph node metastasis in vulvar squamous cell sarcoma patients. Similarly, elevated NLR, along with other inflammation based scores such as PLR, is associated with poorer outcomes in gastric cancer. A meta-analysis by Kim et al^[50]^ found that the combined hazard ratio of mortality was markedly higher in GC patients with elevated NLR than in patients with normal NLR, and patients with higher C-reactive protein, NLR, and Glasgow prognostic score (GPS/mGPS) had lower overall survival. The meta-analysis showed that collectively, these inflammatory markers are prognostic for GC outcomes regardless of country, quality of study, cancer stage, study design, or the inclusion of patients with no-or adjuvant chemotherapy.

Additionally, Miyamoto et al^[8]^ found that their high NLR group had worse preoperative symptoms, postoperative complications (greater than Clavein-Dindo III), intraoperative blood loss, intraoperative transfusion requirement, and median disease free survival. It is evident that preoperative NLR is not only a predictor of short term outcomes (including perioperative complications), but of cancer recurrence, impacting follow up. Li et al^[51]^ describe this as the product of the extended inflammatory postoperative state, which then leads to an extended period of immunosuppression, facilitating micrometastatic spread.

Interestingly, Ishiziuka et al^[52]^ found no relationship between **C**ombination **O**f **P**LR and **N**LR and the levels of tumor markers, for example, CEA,^[53]^ and Ca199.^[54]^ It is not unusual for patients with advanced GC to have tumor marker levels within normal ranges, and such patients would benefit from postoperative surveillance using an inflammation based score such as NLR. The same study found that age, tumor type, lymph node metastasis, and the serum level of albumin are closely associated with the postoperative survival of patients with gastric cancer. Therefore, these other inflammation based factors, along with NLR, may also be useful in the prognostication of gastric cancer.

### 4.3 Elevated preoperative NLR is associated with lymph node metastasis, which could be a valuable adjunct to the current staging pathway

Patients with gastric cancer are staged via a variety of invasive and non-invasive modalities according to the American Joint Committee for Cancer (AJCC) 8th edition guidelines, summarized in Appendix 4 and 5 (Supplemental Digital Content Appendix 4: TNM staging of gastric cancer as per the AJCC Guidelines, 8th edition; and Supplemental Digital Content Appendix 5: Anatomic stage/prognostic groups as per AJCC, 8th edition). There is significant overall variability in these techniques in terms of sensitivity, specificity, cost, and consistency.

Computed tomography remains the gold standard in evaluating nodal status, but can be costly, invasive and inconsistent in accuracy.^[55,56]^ In our experience, finally, nodal metastases are often found that are not enlarged as per CT criteria, that is, nodes <10 mm in diameter. CT is often combined with fluoro-deoxyglucose positron emission tomography (FDG-PET). FDG PET-CT is utilized in esophageal cancer due to its high diagnostic sensitivity for the primary tumor,^[57]^ nodal status,^[58]^ and detection of distant metastases.^[59,60]^ This is crucial for identifying patients with occult metastatic or advanced locoregional disease that would benefit from neoadjuvant chemotherapy. The published sensitivity of FDG PET-CT in staging gastric adenocarcinoma ranges from 60% to 94%.^[61]^ For example, Bosch et al^[62]^ in a retrospective series an additional 16% of their patients to have occult metastases (distant lymph nodes or solid organ disease) detected solely via FDG PET-CT.

In primary GC tumor detection, the sensitivity of FDG-PET is greater for the intestinal histological subtype, and lowest for the sig histology. Kodou et al^[60]^ found that FDG uptake in the primary tumor and lymph nodes was significantly lower with the Sig histology, and sensitivities for lymph node metastasis in Stage III and IV disease were also lower in GC with the Sig histology. By comparison, greater avidity of both primary and metastatic intestinal subtype gastric tumors results in a FDG-PET sensitivity of <50%^[63–65]^ as the spatial resolution of FDG-PET-CT cannot differentiate the primary tumor from positive perigastric nodes.^[64]^ The same study also found that FDG uptake in the primary tumor or lymph nodes was independently associated with Stage III or IV GC, suggesting a more aggressive phenotype. This is explained by Bosch et al,^[62]^ who also found that a significant portion of tumors have no FDG uptake, so no staging information is added in this subset of patients, despite the average size of 18F-FDG negative tumors in their study being 40 mm, with the smallest tumor measuring 10 mm. Additionally, physiological or inflammatory uptake in non-malignant gastric mucosa can obscure a gastric cancer and provide difficulty with primary tumor identification, limiting the specificity of PET-CT.^[66]^

Other more invasive staging methods, such as endoscopic ultrasound, are also utilized. Meta-analysis by Chen et al^[56]^ showed a relatively high sensitivity of EUS for gastric cancer N staging (82%), but lower specificity (68%). However, this method is invasive, and operator dependent. NLR as an adjunct nodal detection method would reflect the inflammatory balance of malignancy.

Preoperative NLR allows accurate staging of nodal status and guides the therapeutic strategy and prognosis,^[46]^ with randomized trials proving the benefit of neoadjuvant chemotherapy (NAC) in patients presenting with advanced disease.^[67]^

The addition of NLR to the staging process has the potential to expand personalized medicine in this field. Stratification of high-risk patients means they can be targeted for intensive staging via the above imaging methods. By pre-treatment detection of nodal disease, patients may be offered neoadjuvant therapy, thus potentially avoiding salvage therapy in the form of adjuvant chemoradiotherapy for intraoperatively detected metastatic disease. Patients with nodal disease can be counseled more fully as to their prognosis, allowing them to make more informed decisions, and undergo tailored surveillance.

Neoadjuvant therapies are effective in controlling LN metastasis in gastric cancer, thereby reducing N stage and increasing complete resection.^[68–70]^ The benefits of neoadjuvant therapy have been analyzed in previous studies,^[71,72]^ with increasingly more studies recommending preoperative therapies for GC patients with nodal involvement.^[73,74]^ In contrast, adjuvant only therapies are regarded as salvage treatments for advanced disease following resection in Western practice, though are often used routinely in Asia. Current ESMO^[75]^ and NCCN^[76]^ guidelines recommend patients with stage II disease or greater undergo neoadjuvant chemotherapy For stage IB GC, the recommendation is for patients to have neoadjuvant chemotherapy if lymph node positive, or chemoradiation if lymph node positive after resection. By comparison, Japanese guidelines^[77]^ recommend patients with Stage II disease and above with lymph node involvement undergo adjuvant chemotherapy, and for stage 2 and above with “bulky” lymph node disease to undergo neoadjuvant therapy. Adjuvant therapy is not easily tolerated by a more debilitated postoperative cohort, particularly in the case of chemoradiotherapy.^[78,79]^ In fact, only almost 50% of patients randomized in the MAGIC and FNCLCC-ACCORD^[80]^ trials completed adjuvant therapy, due to early death after surgery, disease progression, postoperative complications, or toxicity.^[81]^ Certainly, this may explain the findings of a meta-analysis by Hu et al^[82]^ showing neoadjuvant chemotherapy as superior in terms of overall survival at 1, 3, and 5 years compared with surgery only, or surgery with adjuvant chemotherapy. Certainly, adjuvant treatment has an important role in the general management of a case, especially in patients with positive nodal disease (ypN) or >50% vital tumor cells after neoadjuvant chemotherapy.^[83]^ There potential benefit in this cohort should be balanced against the risk of chemotherapy related effects. Additionally, regarding chemoradiotherapy, a meta-analysis of 13 randomized clinical trials including nearly 3000 patients did not find any difference in outcomes between neoadjuvant and adjuvant chemoradiotherapy.^[84]^

Additionally, minimally invasive procedures such as endoscopic submucosal dissection (ESD), and endoscopic mucosal dissection (EMD) are becoming increasingly frequent in early gastric cancer without lymph node metastasis,^[85]^ as they are physiologically less stressful, resulting in a shorter postoperative hospital stay and has a lower postoperative morbidity rate. There was no difference in survival between the 2 methods in a study comparing curative ESD/EMD and gastrectomy.^[85]^ The major risk of ESD is that pathological N status is not determined, because lymph node resection is not performed.^[86]^ Early gastric cancer is resected specifically for lymph node resection, as the risk of lymph node metastases is 2% and 5%.^[87]^ Determining N stage is also valuable in selecting an appropriate surgical method, given that the degree of lymph node metastasis will influence the course of management.

The approach to lymph node dissection varies, with debate over the extent of lymphadenectomy by comparison of D1, D1+, and D2 resections. Currently, the 8th edition of the UICC/AJCC staging system for gastric cancer recommends the removal of at least 16 lymph nodes (D1+ resection) for correct lymph node assessment.^[88]^ Previous randomized trials have shown that D2 lymphadenectomy was related to a higher possibility of reoperation, morbidity, and mortality, and no prolonged survival was observed compared with D1+ lymphadenectomy.^[89,90]^ However these are older studies where splenectomy and often distal pancreatectomy was included in the resection. Current Japanese guidelines now avoid dissection of station 10 (perisplenic) lymph nodes, unless the greater curve is involved.^[91]^ Another RCT suggested that D2 resection may be more suitable than D1 resection for advanced gastric cancer patients with LN metastasis.^[92]^ High likelihood of node negative disease through conventional staging and the addition of NLR means that more extensive lymphadenectomy could be avoided, with ESD, EMD, or D1 lymphadenectomy used to achieve curative resection. Combined with neoadjuvant therapy, the addition of NLR to preoperative staging will allow accurate staging of LN status prior to surgery, and therefore allocation of patients to an appropriate personalized therapeutic pathway.

Finally, survival analysis by Miyamoto et al^[8]^ based on cancer related prognostic factors, for example, serum CEA, differentiation, and stage, found that elevated NLR is also associated with a reduced survival in gastric cancer. Detection of distant metastatic disease through NLR and appropriate diagnostic imaging might allow the patients to commence palliative therapies sooner, and reduce the risks, complications, trauma, and costs (financial, physical, and psychological) of well-intentioned, but ultimately futile treatment

### 4.4 Several score models have been developed to detect LN metastasis in gastric cancer

For example, Li et al^[93]^ described a nodal status predictive score system in pT2 stage gastric cancer and similarly, Shida et al^[94]^ also recommended a preoperative score system to predict LN metastasis in early gastric cancer. Only tumor-related factors were analyzed and the accuracy, specificity, and sensitivity rates of the model were only 70%, 61.6%, and 63.2% respectively. Comparatively, Pang et al^[28]^ included variables such as tumor size, macroscopic type, depth of invasion, PLR, and NLR predicted LN metastasis with a sensitivity of 82.7% and a specificity of 72.4%. The positive and negative predictive values of the model were 88.7% and 61.5% respectively, and unlike EUS, which is operator dependent, models such as these can be combined with traditional imaging protocols to decrease false positive results, especially for understaged patients who could benefit from adjuvant or neoadjuvant treatment. Further studies are needed in this area, including the development of a stratification score validated in a large cohort of patients.

### 4.5 Limitations

This metanalysis focused on retrospective cohort studies that are prone to faults, including that of publication bias:

- Studies did not specify how they eliminated or reduced the rate of false positive, some tumors may have been misclassified by histology or location.
- Only included English articles, and small studies with cumulative results were not published, leading to potential bias.

The authors of each manuscript mathematically derived their cut-off value for NLR based on their population data, with high variability noted in the optimal NLR cut-off, between 1^[10]^ to 3.5.^[27]^ The translation of the NLR into the clinical setting remains challenging, as the strength of association between NLR and all outcomes, including overall survival, varies between studies. A study by Howard et al^[47]^ found that average values of NLR varies between subgroups of the population, and the magnitude of association between high NLR and survival is greater for certain patients—significantly higher as a baseline in white patients, male patients, over 60s, patients with stage IV disease, and patients with ovarian or pancreatic cancer. Some of these have been addressed in the literature, for example, existing observations of benign ethnic neutropenia, and decreasing immune function with age. However, the same study notes that the assumption that the NLR has equal prognostic value for all patients regardless of demographic factors or clinical characteristics of the disease is highly unlikely to be correct. Similarly, Vano et al^[34]^ found that their optimal NLR cut-off value was highly variable based on the time at which survival was assessed and the indices used for optimality assessment, bringing into question the application of such a cut-off across different populations.

Further prospective, randomized controlled studies are needed to further investigate the role of NLR in N staging and also in the post-treatment phase, such as in the monitoring of response to chemotherapy and prognostic assessment.

Finally, it is important to remember that inflammatory markers are influenced by a number of other factors, for example, age, nutritional status, adjuvant therapy, concurrent infective process, and thus can be falsely elevated.

## 5 Conclusions

This meta-analysis shows an association between NLR and positive lymph node status in gastric cancer patients. As a simple, inexpensive preoperative investigation, this would be a valuable addition to the existing diagnostic pathway, as it can stage patients with LN positive disease, and further identify patients who may benefit from neoadjuvant therapies according to current guidelines. Patients with high NLR should be recognized as a high risk group in terms of recurrence, and should be monitored closely in follow-up.

## Author contributions

**Conceptualization:** Animesh Singla, Neil Merrett, Philip Townend

**Data curation:** Animesh Singla, Krishna Kotecha, Neil Merrett, Philip Townend

**Formal analysis:** Animesh Singla, Krishna Kotecha, Philip Townend

**Investigation:** Animesh Singla, Krishna Kotecha

**Methodology:** Animesh Singla, Philip Townend

**Project administration:** Krishna Kotecha, Philip Townend

**Resources:** Krishna Kotecha

**Software:** Krishna Kotecha

**Supervision:** Neil Merrett

**Validation:** Neil Merrett

**Visualization:** Neil Merrett, Philip Townend

**Writing – original draft:** Krishna Kotecha

**Writing – review & editing:** Animesh Singla, Neil Merrett, Philip Townend

## Supplementary Material

Supplemental Digital Content

## Supplementary Material

Supplemental Digital Content

## Supplementary Material

Supplemental Digital Content

## Supplementary Material

Supplemental Digital Content

## Supplementary Material

Supplemental Digital Content
