# Supplementary material for: Association between neutrophil-lymphocyte ratio and lymph node metastasis in gastric cancer: A meta-analysis
Source: Medicine (Baltimore). 2022 Jun 24;101(25):e29300. doi: 10.1097/MD.0000000000029300 (PMC9276313; doi:10.1097/MD.0000000000029300)
Supplement: Supplemental Digital Content [file medi-101-e29300-s001.docx]

|  | Selection | | | | Comparability | | Outcome | | |  |
| --- | --- | --- | --- | --- | --- | --- | --- | --- | --- | --- |
| Study | Representativeness of exposed cohort | Selection of non exposed cohort | Ascertainment of exposure | Demonstration that outcome of interest not present at start of study | Adjust for the most important risk factors | Adjusts for other risk factors | Assessment of outcome | Follow-up length | Loss of follow-up rate | **Total Quality score** |
| Zhang LX | 1 | 1 | 1 | 0 | 0 | 1 | 1 | 1 | 1 | **7** |
| Pang W | 1 | 1 | 1 | 0 | 1 | 1 | 1 | 1 | 0 | **7** |
| Song S | 1 | 1 | 1 | 0 | 0 | 1 | 1 | 0 | 0 | **5** |
| Yu L | 1 | 1 | 1 | 0 | 1 | 1 | 1 | 1 | 1 | **8** |
| Kim EY | 1 | 1 | 1 | 0 | 0 | 1 | 1 | 1 | 0 | **6** |
| Jiang N | 1 | 1 | 1 | 0 | 0 | 1 | 1 | 1 | 0 | **6** |
| Hsu JT | 1 | 1 | 1 | 0 | 0 | 1 | 1 | 1 | 0 | **6** |
| Ubukata H | 1 | 1 | 1 | 0 | 0 | 0 | 1 | 1 | 1 | **6** |
| Shimada H | 1 | 1 | 1 | 0 | 0 | 0 | 1 | 1 | 0 | **5** |
| Zhang Y | 1 | 1 | 1 | 0 | 0 | 1 | 1 | 1 | 1 | **7** |
| Mori M | 1 | 1 | 1 | 0 | 0 | 1 | 1 | 1 | 0 | **6** |
| Kosuga T | 1 | 1 | 1 | 0 | 1 | 1 | 1 | 1 | 0 | **7** |

Supplemental Digital Content (Appendix 1): Newcastle Ottawa Scale scoring system of study quality
