# Supplementary material for: Association between neutrophil-lymphocyte ratio and lymph node metastasis in gastric cancer: A meta-analysis
Source: Medicine (Baltimore). 2022 Jun 24;101(25):e29300. doi: 10.1097/MD.0000000000029300 (PMC9276313; doi:10.1097/MD.0000000000029300)
Supplement: Supplemental Digital Content [file medi-101-e29300-s002.docx]

Supplemental Digital Content (Appendix 2) – Data extracted from studies

Zhang LX

|  | Lymph Node positive | Lymph node negative | Total |
| --- | --- | --- | --- |
| High NLR | 330 | 183 | 513 |
| Low NLR | 218 | 173 | 391 |
| Total | 548 | 356 | 904 |

Pang W

|  | Lymph Node positive | Lymph node negative | Total |
| --- | --- | --- | --- |
| High NLR | 282 | 80 | 362 |
| Low NLR | 74 | 56 | 130 |
| Total | 356 | 136 | 492 |

Song S

|  | Lymph Node positive | Lymph node negative | Total |
| --- | --- | --- | --- |
| High NLR | 763 | 209 | 972 |
| Low NLR | 767 | 251 | 1018 |
| Total | 1530 | 460 | 1990 |

Yu L

|  | Lymph Node positive | Lymph node negative | Total |
| --- | --- | --- | --- |
| High NLR | 113 | 18 | 131 |
| Low NLR | 123 | 37 | 160 |
| Total | 236 | 55 | 291 |

KIM

|  | Lymph Node positive | Lymph node negative | Total |
| --- | --- | --- | --- |
| High NLR | 298 | 441 | 739 |
| Low NLR | 397 | 850 | 1247 |
| Total | 695 | 1291 | 1986 |

Jiang

|  | Lymph Node positive | Lymph node negative | Total |
| --- | --- | --- | --- |
| High NLR | 216 | 93 | 309 |
| Low NLR | 38 | 30 | 68 |
| Total | 254 | 123 | 377 |

Hsu

|  | Lymph Node positive | Lymph node negative | Total |
| --- | --- | --- | --- |
| High NLR | 173 | 84 | 257 |
| Low NLR | 472 | 301 | 773 |
| Total | 645 | 385 | 1030 |

Ubukata H

|  | Lymph Node positive | Lymph node negative | Total |
| --- | --- | --- | --- |
| High NLR | 60 | 10 | 70 |
| Low NLR | 35 | 52 | 87 |
| Total | 95 | 62 | 157 |

Shimada H

|  | Lymph Node positive | Lymph node negative | Total |
| --- | --- | --- | --- |
| High NLR | 76 | 51 | 127 |
| Low NLR | 377 | 524 | 901 |
| Total | 453 | 575 | 1028 |

Zhang Y

|  | Lymph Node positive | Lymph node negative | Total |
| --- | --- | --- | --- |
| High NLR | 84 | 14 | 98 |
| Low NLR | 59 | 25 | 84 |
| Total | 143 | 39 | 182 |

Mori et al

|  | Lymph Node positive | Lymph node negative | Total |
| --- | --- | --- | --- |
| High NLR | 46 | 4 | 50 |
| Low NLR | 40 | 10 | 50 |
| Total | 86 | 14 | 100 |

Kosuga et al

|  | Lymph Node positive | Lymph node negative | Total |
| --- | --- | --- | --- |
| High NLR | 73 | 43 | 116 |
| Low NLR | 13 | 23 | 36 |
| Total | 86 | 66 | 152 |
