# Supplementary material for: Association between neutrophil-lymphocyte ratio and lymph node metastasis in gastric cancer: A meta-analysis
Source: Medicine (Baltimore). 2022 Jun 24;101(25):e29300. doi: 10.1097/MD.0000000000029300 (PMC9276313; doi:10.1097/MD.0000000000029300)
Supplement: Supplemental Digital Content [file medi-101-e29300-s003.docx]

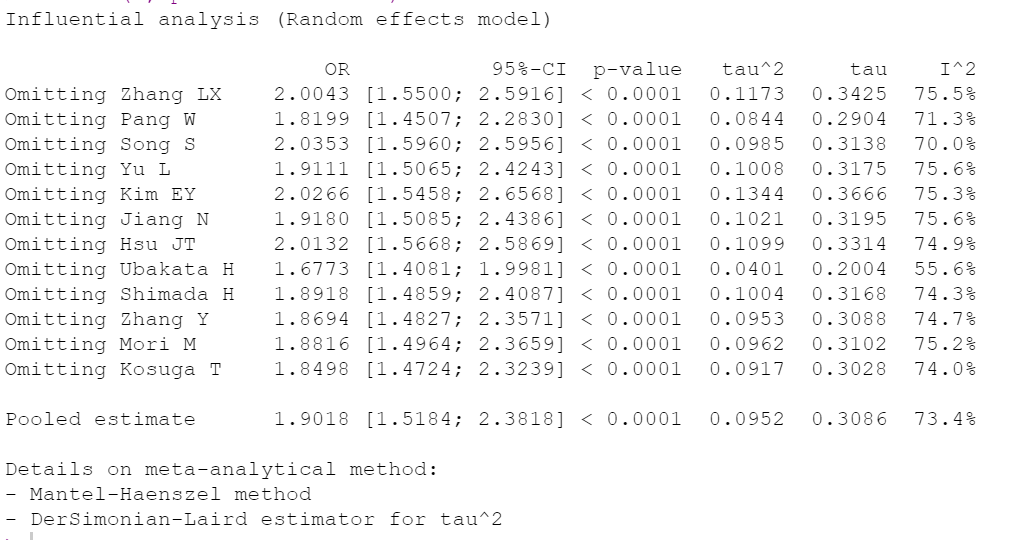


**Supplemental Digital Content (Appendix 3):**

**Exclusion analysis** shows that the paper by Ubakata et al contributed most to the heterogeneity. With exclusion of this paper, the OR remained 1.67 (95% CI of 1.41- 2.00)
