# Supplementary material for: Association between neutrophil-lymphocyte ratio and lymph node metastasis in gastric cancer: A meta-analysis
Source: Medicine (Baltimore). 2022 Jun 24;101(25):e29300. doi: 10.1097/MD.0000000000029300 (PMC9276313; doi:10.1097/MD.0000000000029300)
Supplement: Supplemental Digital Content [file medi-101-e29300-s004.docx]

| Primary tumour (T) | | Regional lymph nodes (N) | | Distant metastasis (M) | |
| --- | --- | --- | --- | --- | --- |
| TX | Primary tumour cannot be assessed | **NX** | Regional lymph node(s) cannot be assessed | **M0** | No distant metastasis |
| T0 | No evidence of primary tumour | **N0** | No regional lymph node metastasis | **M1** | Distant metastasis or positive peritoneal cytology |
| Tis | Carcinoma *in situ*: intraepithelial tumour without invasion of the lamina propria | **N1** | Metastasis in 1–2 regional lymph nodes |  |  |
| T1a | Tumour invades the lamina propria or the muscularis mucosae | **N2** | Metastasis in 3–6 regional lymph nodes |  |  |
| T1b | Tumour invades the submucosa | **N3** | Metastasis in 7 or more regional lymph nodes |  |  |
| T2 | Tumour invades the muscularis propria | **N3a** | Metastasis in 7–15 regional lymph nodes |  |  |
| T3 | Tumour penetrates the subserosal connective tissue without invasion of the visceral peritoneum or adjacent structures^a^ | **N3b** | Metastasis in 16 or more regional lymph nodes |  |  |
| T4 | Tumour invades the serosa (visceral peritoneum) or adjacent structures^b^ |  |  |  |  |
| T4a | Tumour invades the serosa (visceral peritoneum) |  |  |  |  |
| T4b | Tumour invades adjacent organs or structures^b^ |  |  |  |  |

1. T3 tumours also include those extending into the gastrocolic or gastrohepatic ligaments, or into the greater or lesser omentum, without perforation of the visceral peritoneum covering these structures
2. Adjacent structures include the spleen, transverse colon, liver, diaphragm, pancreas, abdominal wall, adrenal gland, kidney, small intestine and retro-peritoneum.

**Supplemental Digital Content (Appendix 4): TNM staging of gastric cancer as per the AJCC Guidelines, 8^th^ edition.^1^**

1. Amin MB, Edge S, Greene F, Byrd DR, Brookland RK, Washington MK, Gershenwald JE, Compton CC, Hess KR, et al. (Eds.). *AJCC Cancer Staging Manual (8th edition).* Springer International Publishing: American Joint Commission on Cancer; 2017 [cited 8^th^ July 2021]
