# Supplementary material for: Association between neutrophil-lymphocyte ratio and lymph node metastasis in gastric cancer: A meta-analysis
Source: Medicine (Baltimore). 2022 Jun 24;101(25):e29300. doi: 10.1097/MD.0000000000029300 (PMC9276313; doi:10.1097/MD.0000000000029300)
Supplement: Supplemental Digital Content [file medi-101-e29300-s005.docx]

| Stage grouping | T stage | N stage | M stage |
| --- | --- | --- | --- |
| Stage 0 | Tis | N0 | M0 |
| Stage IA | T1 | N0 | M0 |
| Stage IB | T2 | N0 | M0 |
|  | T1 | N1 | M0 |
| Stage IIA | T3 | N0 | M0 |
|  | T2 | N1 | M0 |
|  | T1 | N2 | M0 |
| Stage IIB | T4a | N0 | M0 |
|  | T3 | N1 | M0 |
|  | T2 | N2 | M0 |
|  | T1 | N3 | M0 |
| Stage IIIA | T3 | N2 | M0 |
|  | T2 | N3 | M0 |
|  | T4a | N2 | M0 |
|  | T4a | N1 | M0 |
|  | T4b | N0 | M0 |
| Stage IIIB | T1 | N3b | M0 |
|  | T2 | N3b | M0 |
|  | T3 | N3a | M0 |
|  | T4a | N3a | M0 |
|  | T4b | N1 | M0 |
|  | T4b | N2 | M0 |
| Stage IIIC | T3 | N3b | M0 |
|  | T4a | N3b | M0 |
|  | T4b | N3a | M0 |
|  | T4b | N3 | M0 |
| Stage IV | Any T | Any N | M1 |

**Supplemental Digital Content (Appendix 5) Anatomic stage/prognostic groups as per AJCC, 8^th^ edition.**
